# A speech corpus of Quechua Collao for automatic dimensional emotion recognition

**DOI:** 10.1038/s41597-022-01855-9

**Published:** 2022-12-24

**Authors:** Rosa Y. G. Paccotacya-Yanque, Candy A. Huanca-Anquise, Judith Escalante-Calcina, Wilber R. Ramos-Lovón, Álvaro E. Cuno-Parari

**Affiliations:** Universidad Nacional de San Agustín de Arequipa, School of Computer Science, Arequipa, Peru

**Keywords:** Computer science, Human behaviour

## Abstract

Automatic speech emotion recognition is an important research topic for human-computer interaction and affective computing. Over ten million people speak the Quechua language throughout South America, and one of the most known variants is the Quechua Collao one. However, this language can be considered a low resource for machine emotion recognition, creating a barrier for Quechua speakers who want to use this technology. Therefore, the contribution of this work is a 15 hours speech corpus in Quechua Collao, which is made publicly available to the research community. The corpus was created from a set of words and sentences explicitly collected for this task, divided into nine categorical emotions: happy, sad, bored, fear, sleepy, calm, excited, angry, and neutral. The annotation was performed on a 5-value discrete scale according to 3 dimensions: valence, arousal, and dominance. To demonstrate the usefulness of the corpus, we have performed speech emotion recognition using machine learning methods and neural networks.

## Abstract (summary)

**Table:** 

Measurement(s)	emotion/affect behavior trait
Technology Type(s)	Hearing audio material
Sample Characteristic - Organism	Homo sapiens
Sample Characteristic - Location	Peru

## Background & Summary

Studies have shown that AI is able to understand and express emotions, improving the quality and effectiveness of Human-Machine Interaction^1,2^. Speech emotion recognition (SER) is useful in affective computing applications such as patient in-home monitoring^3^, early detection of psychiatric diseases and disorders^4–6^, support diagnosis and treatment in military healthcare^7^, recognize deceptive speech^8,9^, or stress on it^10,11^. Furthermore, SER is used in call center conversations to categorize voice mail messages. Humans naturally can recognize emotions from facial expressions and speech. Experts claim that facial expressions are universal. However, speech is not; there are variations in the vocal signature for some emotions, e.g., anger. This situation is due to differences in the expression of emotions in different cultures and languages^12,13^.

Quechua is spoken in seven South American countries (Peru, Ecuador, Colombia, Bolivia, Argentina, Chile, and Brazil). In Peru, it is considered a vital language because it was spoken before the diffusion of the Spanish language, and it is used across the country. However, many Quechua variants are in danger, given that there is a significant decrease in the importance of this language^14^. That is reflected in the lack of Quechua speech datasets annotated with emotions when compared, for example, to Spanish or English. Languages with data scarcity are known as low-resource languages (LRLs)^15^; in this sense, Quechua is a low-resource language.

To the best of our knowledge, no work in the literature focuses on creating a corpus in Quechua for SER. However, few proposals involve the creation of speech corpora for Quechua that deal mainly with automatic speech recognition (ASR). One of these works is Siminchik^16^, a speech corpus for the preservation of Southern Quechua, intended to be used in speech recognition systems. It was created from radio programs and volunteers (native Quechua speakers) who made audio transcriptions. A Hidden Markov Model was used to perform ASR. Although Siminchik has a larger version^17^ that was created by adding more data sources such as recordings of repetitions of audio prompts, readings of text prompts, and free speeches of native speakers, it still seems to be a work in progress (https://www.siminchikkunarayku.pe)^18^. Also, Chacca *et al*.^19^ used Mel-Frequency Cepstral Coefficients (MFCC), Dynamic Time Warping (DTW), and K-Nearest Neighbor (KNN) in an ASR system to recognize numbers in Quechua. For this, isolated words were recorded from 30 native speakers, between men and women, counting from 1 to 10. However, this database was limited as it contained only 300 audios.

Therefore, there is an emotion recognition gap for Quechua speakers. We aim to fill this gap by creating a speech corpus of Quechua Collao for automatic emotion recognition, evaluating its usefulness using machine learning techniques and deep neural networks, and making it publicly available. We selected the dimensional approach, based on valence, arousal, and dominance proposed by Mehrabian and Russell^20^ to indicate people’s state of feeling, as categorical emotions can be recovered from dimensional values^21^. Evaluation with machine learning techniques and neural networks will be helpful as a baseline for other researchers who want to study the recognition of speech emotions with supervised and unsupervised algorithms. Therefore, the creation of this corpus not only fills the emotion recognition gap for Quechua speakers but also opens the opportunity for new studies.

## Methods

### Script creation

Our script comprises 378 words and 1692 sentences, making a total of 2070 instances. These were extracted from different texts written in Quechua Collao, while most of them include a Spanish translation^22–36^. The complete script is divided into nine parts, each consisting of 42 words and 188 sentences. These parts correspond to 9 categorical emotions: happy, sad, bored, fear, sleepy, calm, excited, angry, and neutral. Most words and sentences were chosen according to the emotions that were used. For example, the sentence “Sumaqmi ñawiyki”, which means “your eyes are pretty”, was selected to represent happy emotion.

The script construction was done in five phases. In the first phase, all the documents from which all the words and sentences were extracted were investigated. The second phase was carried out by three people who built the script. These first two phases were carried out by people who speak Spanish natively but do not speak any variant of Quechua. In the third phase, an expert in the Quechua Collao language reviewed and corrected the entire script and provided other sources to replace some sentences per emotion. The fourth phase consisted of selecting these new sentences and replacing some old ones. Finally, the fifth phase was accomplished by a second Quechua language expert, who translated sentences from Spanish into Quechua Collao and made corrections to the entire script.

### Recording sessions

The recording sessions were performed with semi-professional microphones and at semi noiseless spaces at the School of Computer Science - UNSA. The actors were mostly mid-age native Quechua speakers that were paid to record. The whole script (2070 instances) was planned to be recorded by each actor of a group of three women and three men in order to have balanced data in terms of gender. However, an actress (Actress 5) could not finish due to unexpected situations, so her work was completed by another actress (Actress 7). Table 1 shows information related to actors.

**Table 1:** Demographical information of actresses and actors.

	Gender	Age	Profession	Birthplace
Actress 1	F	43	Quechua Instructor	Puno
Actor 2	M	36	Quechua Instructor	Puno
Actress 3	F	49	Quechua Instructor	Cusco
Actor 4	M	28	Quechua Instructor	Cusco
Actress 5	F	45	Quechua Professor	Cusco
Actor 6	M	36	Quechua Instructor	Apurimac
Actress 7	F	24	Quechua Instructor	Puno

A session consists of one emotion’s recording per actor (230 instances). Previous to the recording, the actors were given the scripts and asked to rehearse with the linguist expert. In each session, the actor/actress is asked to read the script while interpreting the target emotion, and its interpretation is evaluated. If the evaluator decides the performance or pronunciation is inadequate, then the recording of the instance is repeated, asking the actor/ actress to remember situations in the past or giving them hypothetical situations so emotions can be elicited. To avoid performance fatigue, each session was divided into five groups of 46 instances, and after recording each group, a pause of around 5 minutes was done.

**Fig. 1:**
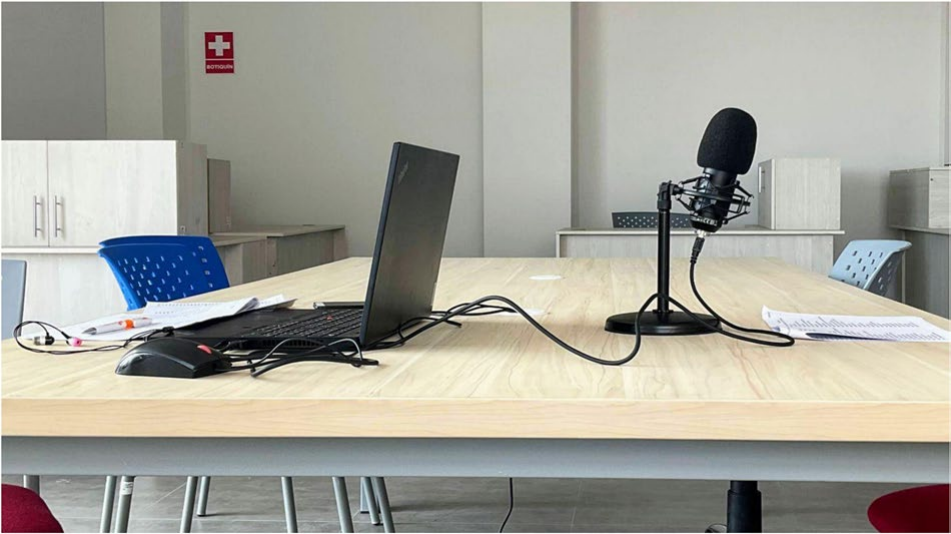
Scene setup for recordings.

The audios were recorded using one of the following microphones: Emita USB Studio Microphone GXT 252 and Blue Snowball USB Microphone. The software used to record was Audacity, with a sample rate 44KHz and one recording channel (mono). The scene setup for recordings is shown in Fig. 1.

### Segmentation

There were two segmentation stages. First, an automatic segmentation was performed using the software Praat. Each WAV file obtained from the recording session was divided into small segments using a script that splits the original audio file after detecting silence. However, this process is not flawless, and a manual segmentation had to be performed. Each audio segment was listened to by a person who verified if it was correct or not. If not, the segment was lengthened or shortened to avoid cutting off words and to delete noise or silence at the beginning or end of the segment. In addition, some fragments were merged when a word or sentence was split incorrectly. After the automatic and manual segmentation, the resulting audios were assigned a random name so they could be labeled without previous information on which emotion they belonged to by the annotators.

### Annotation

Two men and two women were employed and paid to annotate the audio labels. They are Quechua Collao native speakers and Quechua instructors, ages ranging from 27 to 46.

Each annotator labeled the 12420 audios over a 4-week period by assigning valence, arousal, and dominance values. Each week, 3105 audios were released to be annotated. It must be noted that we only considered five days a week dedicated to annotation. A methodology was recommended to ensure the annotation quality, suggesting about 6 hours a day to annotate, preferably in two 3-hour groups, while taking a 10-minute pause after 1.5 hours. During the last week, one of the female annotators had to be replaced by another female Quechua instructor due to some external inconveniences.

**Fig. 2:**
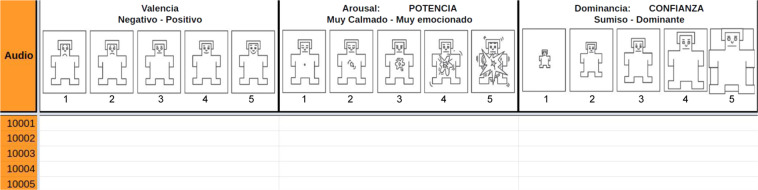
Example of the sheet used by the annotators to label the audios.

The annotation process was performed using a sheet for each annotator, where they had to write the valence, arousal, and dominance values for each audio. A scale of 1 to 5 was used, and visual aid was provided using self-assessment manikins (SAMs)^37^. Figure 2 shows an example of the sheets used for annotation, where the first column shows the audio filename, and the last three columns correspond to valence, arousal, and dominance. In the header, 5 SAMs are shown for each emotional dimension, with a brief description in Spanish: Negative - Positive for valence, Very calm - Very excited for arousal, and Submissive - Dominant for dominance.

### Ethics declaration

All procedures performed in this study were approved by the KUSISQA project research committee in accordance with the ethical standards of the National University of San Agustin. Likewise, individual written informed consent was obtained from all participants involved in the study, where the actors and actresses were informed that their voices would be freely shared anonymously.

## Data Records

The corpus is publicly available at Figshare^38^. It contains 15 h 15 min of audio divided into 12420 segments of audio. Table 2 summarizes its content.

**Table 2:** Corpus^38^ summary.

Corpus features	
Number of audio segments	12420
Total segments length	15 h 15 m 15 s
Dimensions	Valence, Arousal, and Dominance
Annotations scale	1, 2, 3, 4, 5
Number of speakers	7
Gender of speakers	4 women and 3 men
Age range of speakers	24–49 years

The corpus^38^ can be downloaded as a zip file that contains 4 directories. All audio segments in WAV format are found in the **Audios** folder; each audio is randomly named by numbers ranging from 10001 to 22420. This action was carried out to avoid bias. Detailed information of each audio is found in a file inside the **Data** folder, in a file named *Data.csv*. This file is made up of five columns, where:

- The *Audio* column contains the name of the WAV files.
- The *Emotion* column represents the categorical emotion of the audio segment.
- The *Actor* column contains the code of the actor who performed and interpreted the audio segment. The code comprises an *a* and a number from 1 to 6 representing the six actors (example: a2).
- The *File* column contains the original name of each audio segment, this name is divided into two segments by an underscore: The first represents the code of the actor, and the second segment is composed of a letter and a number; the letter represents the emotion (H = Happy, T = Sad, B = Bored, F = Fear, S = Sleepy, C = Calm, E = Excited, A = Angry, and N = Neutral) and the number represents the position of the audio segment. For example, the name a2_B159.wav indicates that the segment belongs to actor 2 (a2), that it belongs to the emotion bored (B), and that it is the 159th segment in this set.
- *Duration (s)* column indicates the duration in seconds of each audio segment.

The annotations for each audio segment are found in the **Labels** folder, and the emotional dimensions of valence are found in the *Valence.csv* file, which is made up of 5 columns: the first contains the name of the audio segment, and the other four store the label made by each annotator. For example, the second column contains the labels made by annotator 1, represented by the code N1. The values for the emotional dimension of arousal and the emotional dimension of dominance are found in the files *Arousal.csv* and *Dominance.csv*, respectively, and have the same structure as the *Valence.csv* file.

The general average of each emotional dimension is found in the *Labels.csv* file, also within the **Labels** folder, where the first column represents the name of the audio segment, the second, third, and fourth columns contain the average of the labels made by the four annotators of the dimensions emotional valence, arousal, and dominance respectively.

Finally, the **Script** folder contains *Script.xlsx*, which is the script used for the corpus^38^ creation. This file comprises 9 sheets for the 9 categorical emotions used. Each word and sentence has an ID that employs the same notation as that of the *File* column of *Data.xlsx*, explained previously, without considering the actor prefix. For example, T159 is an ID that corresponds to the 159th sentence of the Sad emotion.

## Technical Validation

Below, we present three validations of our corpus^38^: Emotional Diversity, Annotation Consensus, and Machine Validation.

### Emotional diversity

**Fig. 3:**
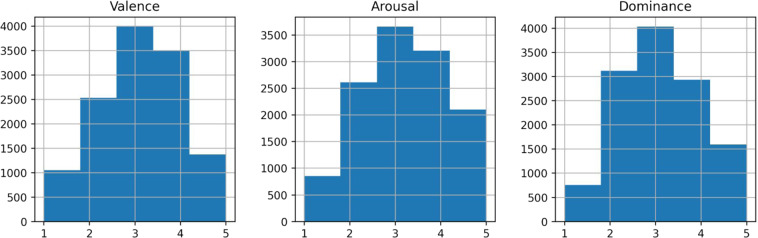
Histograms for each dimensional attribute in the Quechua Collao corpus.

To evaluate the emotional diversity, we created histograms for each dimension that depict instances’ distribution in labels from 1 to 5 (Fig. 3). As can be seen, the corpus^38^ has an unbalanced emotional content (valence - arousal), a common problem in most data sets for SER^39^. Data imbalance is also observed for dominance. This problem could be solved by adding more annotators.

### Annotation consensus

The reliability of the labels between annotators was calculated using Cronbach alpha coefficients. Table 3 shows the results; as can be seen, there is a higher agreement for arousal and dominance than for valence. It must be noted that all 12420 audios were used to calculate the Cronbach alpha coefficients.

**Table 3:** Cronbach’s alpha coefficients.

Valence	Arousal	Dominance
0.513	0.619	0.625

### Machine validation

To validate the usefulness of this Quechua Collao corpus^38^, experiments for dimensional emotion recognition were carried out using machine learning methods and neural networks. These experiments establish a baseline standard for future research.

The machine learning (ML) methods used are Support Vector Regression (SVR), K-neighbors Regression (KNR), and Random Forest Regression (RFR). They were implemented using Scikit-Learn^40^.

The neural network models used are Multilayer Perceptron (MLP), Long Short-Term Memory (LSTM) network, and Convolutional Neural Network (CNN), whose implementation details can be found in Atmaja and Akagi’s work^41^. The hyperparameters were adjusted to the Quechua Collao corpus^38^ for each model.

All the parameters for these methods were obtained after extensive testing to optimize the mean of the Concordance Correlation Coefficient (CCC)^42^ scores for valence, arousal, and dominance.

Recall that the final label for each audio track was calculated as the average of the annotators’ individual labels.

A feature vector was obtained from each audio by using the Geneva Minimalistic Acoustic Parameter Set (GeMAPS)^43^ and the procedure done by Atmaja and Akagi^41^. Twenty-three low-level descriptors (LLDs), presented in Table 4, were computed on a frame-based level. Mean and standard deviation were calculated over the feature set to attain a vector with 46 elements for each audio.

**Table 4:** GeMAPS low-level descriptors (LLDs) and feature vector structure.

**LLDs**	Intensity, alpha ratio, hammarberg index, spectral slope 0–500 Hz, spectral slope 500–1500 Hz, spectral flux, 4 MFCCs, *f*_*o*_, jitter, shimmer, Harmonics-to-Noise Ratio (HNR), Harmonic difference H1-H2, Harmonic differ- ence H1-A3, F1, F1 bandwidth, F1 amplitude, F2, F2 amplitude, F3, and F3 amplitude.
**Feature vector**	Mean (of LLDs), std (of LLDs).

Adapted from Atmaja and Akagi’s work^41^.

After obtaining the feature vectors for all audios, they were separated into three sets: 60% for training, 20% for validation, and 20% for testing. All methods use these sets. Their results are measured using the CCC criteria.

#### K-Neighbors regressor

KNR is the regression based on k-nearest neighbors (KNN). Since KNN has been used in several SER categorical challenges^44–46^. In this case, we used KNR to validate a multidimensional problem since we need to intuitively approximate the association between variables and the continuous result. For the implementation we used the Sklearn library and the following parameters: *8* as the number of neighbors, *distance* as the weight function, *Minkowski* as the distance metric and *1* as the power parameter.

#### Support vector regressor

SVR is a widely used method in approaches to tackle SER^47–49^, as well as in SER challenges, like the AudioVisual Emotion Challenge (AVEC), to establish a baseline^50^.

We used the Nu Support Vector Regression (NuSVR) implementation with the following parameters: *nu* was set to 1, *C* was 172600, the kernel was rbf and *gamma* was *scale*.

#### Random forest regressor

A Random Forest is an estimator that works by averaging the prediction of different decision trees. RFR has been used in different works of SER^51–53^, and also as a baseline for a new audio-visual dataset^54^. For the implementation, we used Sklearn with 100 trees.

#### Multilayer perceptron

The MLP model was trained with five layers of 256,128, 64, 32, and 16 nodes per layer and ReLU activations. The optimizer was Adam, with a learning rate of 0.001 and a batch size of 32.

#### Long short-term memory network

The LSTM model used an Adam optimizer, a batch size of 32 and 3 layers with 512, 256, and 128 nodes.

#### Convolutional neural network

The CNN used for recognizing speech emotions is made up of 5 layers. The first layer uses 256 neurons, and these are reduced by half in each of the subsequent layers (256, 128, 64, 32, 16). The activation function ReLU was used in the five layers, and the batch size used was 32. An Adam optimizer is used to adjust the learning rate during the training process.

The MLP, CNN, and LSTM models were trained for a maximum of 180 epochs with an early stopping with patience of 10 epochs monitored on the validation loss.

The CCC scores for the ML methods are presented in Table 5. RFR has the best mean on the CCC scores for each dimension, and arousal and dominance have higher CCC scores than valence.

**Table 5:** Results of CCC scores for ML methods.

Method	Validation set				Test set			
	Valence	Arousal	Dominance	Mean	Valence	Arousal	Dominance	Mean
SVR	0.401	0.646	0.698	0.582	0.427	0.625	0.684	0.578
KNR	0.324	0.479	0.520	0.441	0.294	0.508	0.538	0.447
RFR	**0.485**	**0.699**	**0.735**	**0.639**	**0.492**	**0.684**	**0.717**	**0.631**

Table 6 shows the CCC scores of the neural network methods. The LSTM model performs better than CNN and MLP in average, while the tendency observed in the ML methods also occurs, as valence has a lower CCC score when compared to arousal and dominance.

**Table 6:** Results of CCC scores for neural network methods.

Method	Validation set				Test set			
	Valence	Arousal	Dominance	Mean	Valence	Arousal	Dominance	Mean
MLP	0.612	0.748	0.769	0.710	0.636	0.756	0.775	0.723
LSTM	**0.627**	0.749	**0.778**	**0.718**	**0.648**	**0.764**	0.782	**0.731**
CNN	0.612	**0.751**	0.766	0.710	0.632	0.747	**0.785**	0.721

These scores were averaged over 10 runs of each algorithm.

In general, neural network methods have better results than ML methods, while CCC scores for valence consistently are lower than those for arousal and dominance. This fact reflects the Cronbach alpha coefficients shown in Table 3, where the valence labels have the lowest agreement among the annotators.

## Usage Notes

The individual labels are also provided along with the averaged ones, making it possible to apply a different method to calculate the final labels for each audio.

The corpus^38^ is built over the variant Collao of Quechua. Thus, this variant has noticeable differences from other variants and should not be used to generalize Quechua.

The main limitation is the emotional imbalance of the corpus^38^, which can lead to low performance in SER algorithms for labels of instances with low frequency. Furthermore, we must note that the recordings are performed in a controlled environment with only six mid-age speakers acting established emotions. Therefore, given these limitations, the recordings provide prototypical insights for studying emotions but they cannot fully represent the emotional expressions of all Quechua speakers that are observed in real life.

## Data Availability

Code and data splits for baseline algorithms are available at Github, in https://github.com/qccData/qccCorpus.
